# Treatment recommendation differences for schizophrenia and major depression: a population-based study in a Vietnamese cohort

**DOI:** 10.1186/s13033-018-0247-6

**Published:** 2018-11-14

**Authors:** Kerem Böge, Eric Hahn, Tien Duc Cao, Lukas Marian Fuchs, Lara Kim Martensen, Georg Schomerus, Michael Dettling, Matthias Angermeyer, Van Tuan Nguyen, Thi Minh Tam Ta

**Affiliations:** 1 Department of Psychiatry and Psychotherapy, Charité University Medicine Berlin, Campus Benjamin Franklin, Hindenburgdamm 30, 12203 Berlin, Germany; 2 Department of Psychiatry and Psychological Medicine, Hospital 103, Military Medical University, Hanoi, Vietnam; 3 Center for Public Mental Health, Untere Zeile 13, 3482 Gösing am Wagram, Austria; 4 Department of Psychiatry, Ernst Moritz Arndt University, Greifswald, Germany; 5 Department of Psychiatry, Hanoi Medical University, Hanoi, Vietnam; 6 Berlin Institute of Health, Berlin, Germany

**Keywords:** Public attitudes, Treatment recommendations, Schizophrenia, Depression, Mental health system, Vietnam

## Abstract

**Background:**

In Vietnam, the mental health care infrastructure is on the verge of transformation with an increase in the demand for access to adequate and effective mental health care services. Public attitudes towards mental illness, as well as corresponding treatment options influence help-seeking behaviors of patients and caregivers, affecting the course of their treatment. This study assesses attitudes towards treatment options for depression and schizophrenia, as the two most common psychiatric disorders in Vietnam, accounting for at least 75% of all psychiatric inpatients.

**Methods:**

A general population-based survey was conducted in Hanoi, Vietnam between April and August 2013. Participants received a description of a person with symptoms of either depression (n = 326) or schizophrenia (n = 403) and were asked to give recommendations for adequate sources of mental health support and treatment options. Multiple analyses on a single item level compared the likelihood of recommendation between schizophrenia and depression.

**Results:**

Overall, respondents recommended health care services, ranging from seeking mental health care professionals, psychotherapists, and psychiatrists for both disorders. Psychotherapy was the most favored treatment method, whereas further treatment options, such as concentration and relaxation exercises, meditation or yoga and psychotropic medication were also endorsed as helpful. For the schizophrenia vignette condition, psychotherapy, visiting a psychiatrist or psychotherapist received stronger endorsement rates as compared to the depression vignette. Furthermore, ECT, Feng Shui-based practices, praying and visiting natural healers were recommended less by respondents for the depression vignette in comparison with the schizophrenia vignette.

**Conclusions:**

The Vietnamese public endorsed evidence-based treatment recommendations from a variety of treatments options. Differences in the treatment recommendations between depression and schizophrenia reflected the perceived severity of each disorder. Further developments of the Vietnamese mental health care system concerning mental health care providers, as well as the legal regulations surrounding the provision of psychotherapy are needed.

## Introduction

Mental disorders are the most common cause of years lived with disability in the world [1]. In 2017, the WHO named depression ‘the single largest contributor to global disability’ [2]. Estimates report that around 300 million people, or at least 4.4% of the world’s population, suffer from depression [2]. The causal effects of depression and schizophrenia on disability, quality of life, and economic situation [1, 3] and the need for timely and adequate treatment was emphasized by the World Health Organization [2].

### The mental health treatment gap in Vietnam

Research and policies concerning the treatment of mental illness focus on the lack of adequate provision of health care services as potential barriers to appropriate and efficient mental health care [4]. Globally, the WHO [4] counts merely 0.71 psychiatric treatment facilities per 100,000 population. This deficit in psychiatric facilities is particularly evident in low- and middle-income countries that however account for the majority of the global disability-adjusted life years due to mental illness. Accordingly, appropriate mental health care resources are especially warranted in those countries [5]. According to the World Bank [6], on the one hand, Vietnam progresses towards reaching upper-middle-income status. On the other hand, the Vietnamese mental healthcare system did not develop on par with the economic progress. In 1999, national strategies to improve mental health care in Vietnam have been implemented, especially in recent years’ promotion of mental health care has received public attention. Strategies developed with the WHO, the National Institute of Mental Health (NIMH) and HMU seek to comprehensively modernize the mental health care and education system, as well as to internationalize the current WHO guidelines under the WHO Mental Health Gap Action Plan (mhGAP, 2013–2020). However, persistent insufficient funding still results in reduced treatment utilisation and a significant treatment gap [3, 6, 7], despite high public mental health demands [8]. Considering that estimates only count 0.63 psychiatrists, 0.1 psychologists, and 6.8 psychiatric hospital beds per 100,000 population, located in 39 mental hospitals, as well as 19 psychiatric units in general hospitals, the treatment services gap, calls for further mental health system reforms [9, 10]. In Vietnam, 14.9% of the population are estimated to suffer from at least one of the ten most common mental disorders, leaving 12 million people in need of mental health care [11]. The most prevalent disorders according to those official numbers were alcohol abuse (5.3%), followed by depression (2.8%) and anxiety (2.6%) with schizophrenia ranking seventh (0.5%) [11]. Among patients treated in psychiatric hospitals in Vietnam, the three most common disorders are schizophrenia (60%), mood disorders (15%) and neurotic disorders (15%) [12]. Since mental health service capacity is influenced by sociocultural barriers related to stigmatisation [9, 13], developing and evaluating culturally appropriate evidence-based treatments [14, 15] remains a challenge for mental health research.

### Help-seeking behaviour and cultural perceptions of mental illness in Vietnam

Public attitudes and explanatory models play a role not only in understanding and conceptualizing mental health, [16, 17] but also in determining which treatment option is chosen in the help-seeking process, the availability of services and mental health literacy [18, 19].

In Vietnam, healthcare is delivered at four levels (province, central, commune and province) by either primary care or community-based services [20]. Studies conducted in Vietnam [21–23] illustrate a strong preference for community-based services, which cover around two-thirds of the population need. The community-based mental health program (CMH) in Vietnam includes a free access to a number of essential psychotropic medicines for prioritized mental disorders such as schizophrenia, depression and epilepsy [11], as well as monthly consultation with the medical staff in the community health stations. Financial and social support are also offered to patients with severe mental disorders and their caregivers. Moreover, the psychiatric private doctors’ offices cover the community-based services in the urban areas. Even though, the CMH is considered as a national health target, only schizophrenia and epilepsy are discussed shortly within the national report, disregarding depression and thus displaying current priorities [7]. In addition to that, Van der Ham et al. [22] conclude that within the Vietnamese population, treatment options besides family care and community-based approaches are often not considered. They attributed this to a lack of mental health literacy and biased perceptions towards mental health care options. In this regard, the notion of public stigma plays a central role. Public stigma refers to negative attitudes towards persons with mental illness that shape beliefs about recommended treatment options [24–26]. In Vietnam, stigma towards mental illness is shaped by multiple factors, such as gender [27], religiosity [27, 28] or urbanity [29, 30]. Research within Vietnam [11, 31] shows high rates of experienced discrimination towards patients diagnosed with schizophrenia, drawing similarity to comparable Asian cohorts such as from China [32] and Western countries [33].

In particular, discrimination has a negative impact on the help-seeking behavior of patients, families and caregivers. Moreover, severe psychiatric disorders such as schizophrenia are still often perceived as dangerous and incurable madness [11]. Another related type of stigma concerns the public perception of healthcare professionals, and psychiatric institutions, which in turn influences treatment recommendations and the perception of patients with mental illness [27, 34, 35].

With schizophrenia and depression being among the two most commonly treated disorders in Vietnamese hospitals [12], this study aimed to understand public perceptions and attitudes of adequate treatment options to those prominent disorders. Previous research [36] assessed treatment recommendations towards schizophrenia, depression and alcohol dependence of lay people in Germany, with particular attention paid to the explicit differentiation of the illness symptoms and expressions. Recent research reported that public stereotypes and stigma towards mental health might change over time [37] and may be reduced through tailored educational efforts [38]. Since public stigmatization in the context of mental health care is associated with the help-seeking behaviour, this study aims as the first—to our knowledge—to explore public understanding concerning evidence-based treatments for two of most commonly treated mental illnesses in Vietnam, namely schizophrenia and depression [12]. Varying views, either traditional or modern, as well as a lack of knowledge have been mentioned as key factors influencing the help-seeking process and mental health perception in Vietnam [22]. However, even though schizophrenia remains highly present and inextricably associated with the concept of psychiatry in Vietnam, an understanding of depression as a mental disease gains prominence [22]. In addition to that, there remains an assumption that traditional psychiatric treatment will be prescribed for schizophrenia, while low-threshold interventions might be selected for depression, ultimately displaying the differences in explanatory models of the two diseases and their adjunct causal beliefs [22]. Furthermore, this study intends to provide an understanding of possible sociocultural barriers to treatment and public mental health literacy in general.

## Methods

### Study participants

The study was conducted between April and August 2013 in Hanoi, an area that is occupied by more than 7,000,000 inhabitants, living in 29 districts (11 urban districts, 18 rural districts) [39]. Participants were recruited through a large personal network of the staff and medical students of the psychiatric department of the Hospital 103, Military Academy of Medicine. Furthermore, the study was approved by the ethics committee of Department of Psychiatry and Psychological Medicine, Hospital 103, Military Academy of Medicine, Hanoi, Vietnam. Only one person per contacted household was allowed to participate and no financial compensation was given. All subjects received written information about the research and its purposes and had to give their signed consent. All returned questionnaires were controlled for systematic errors by the staff of Hospital 103 as well as the second and last author (EH and TT). In total, more than 1000 questionnaires were distributed equally attached with either a vignette illustrating symptoms of schizophrenia or depression. After carefully examining returned questionnaires by respondents for errors and missing data, a total number of 729 respondents, with 403 for schizophrenia and 326 for depression, remained and were considered for the analysis. According to the quota sampling approach the present sample (729) and each vignette subgroup (S = 403, D = 326) were representative and further showed statistically no significant differences between conditions for gender, age, urbanity, household size and unemployment rate (see Table 1), following the Hanoi Census (2009) and the more recent micro-census (2013) which corresponds to the timeframe of sampling.

**Table 1:** Socio-demographic characteristics of survey sample in percent in comparison to the total population of Vietnam and Hanoi municipality according to the census of 2013/2009

Sociodemographic data	Total population of Hanoi (*n *= 6,936,900)	Total survey sample (*n *= 729)	Survey sample schizophrenia (*n *= 403)	Survey sample depression (*n *= 326)
Gender (%)^a^				
Male	49.8	49.5	49.4	49.7*
Female	50.2	50.5	50.6	50.3*
Urbanity (%)^a^				
Urban	42.5	47.9	49.9	45.4
Rural	57.5	52.1	50.1	54.6
Age, years (%)^b^				
15–19	14.9	15.5	15.4^e^	15.6*
20–24	14.0	14.5	15.1	13.8*
25–29	13.0	14.4	13.6	15.3*
30–34	11.4	12.6	12.9	12.3*
35–39	11.1	11.7	12.9	10.1*
40–44	10.2	12.2	12.2	12.3*
45–49	9.2	8.5	7.9	9.2*
50–54	7.7	7.0	7.7	6.1*
55–59	5.2	2.2	1.5	3.1*
60–64	3.3	1.4	0.7	2.1*
Household size (%)^b^				
1	9.1	7.7	6.9	8.6*
2–4	70.2	61	60.8	61.3*
5–6	18.7	25.4	26.8	23.6*
7+	2.0	5.9	5.5	6.4*
Unemployment rate (%)^c^				
Total	2.5	2.5	2.7	2.1
Urban	5.1	2.6	2.0	3.4
Rural	1.6	2.4	3.5	1.1
School education (%)^d^				
Lower secondary school	33.3	22.2	22.3	22.1*
Upper secondary school	66.7	77.8	77.7	77.9*

* Chi squared test: not significant differences between both vignettes for gender, age, school education and household size

^a^Data from micro-census 2013 [39]

^b^Data from full census 2009 [39]

^c^Report on Labour force survey 2013 [40]

^d^Education for All 2015 National Review Report: Viet Nam [41]

^e^Only person ≥ 16 years were allowed to complete the questionnaire

### Questionnaires

Every participant randomly received an unlabelled vignette depicting a person with ICD-10 symptoms of either schizophrenia or major depression. These vignettes have been used and reaffirmed in several studies before [36, 42]. After reading the vignette scenario, respondents were given identical questionnaires asking for their treatment recommendations for the depicted person. Respondents were asked first, which treatment measure they would recommend to the illustrated person and second, to indicate which person or institution they advise for seeking help. For both parts, several choices were given, and the respondents had to indicate their level of agreement with each option on a five-point Likert scale with anchor points at (1) ‘I would strongly advise it’ to (5) ‘I would strongly advise against it’. Therewith, lower mean scores illustrate stronger recommendation rates. Possible items for treatment measures possibilities consisted of commonly endorsed interventions such as psychotherapy, natural medicine, concentration and relaxation exercises, psychopharmacology, meditation and yoga, ECT (electroconvulsive therapy), and the cultural adapted measure for Vietnamese context such as bringing life in balance with Feng Shui rules and praying in a pagoda or any other place of worship. In detail, options for personal and institutional help-seeking were: participation in self-help groups, a general practitioner, a rehabilitation facility, a psychotherapist, the health authority or regional health department, a priest, a psychiatrist, a natural health healer, a person of trust, doing something by oneself, the internet, getting in contact with ancestors via a medium or consult a Feng Shui master, which are common in the Vietnamese context.

### Translation procedure

Both vignettes, as well as the questionnaire, were translated and adapted from German to Vietnamese by the last author who is a native speaker of Vietnamese and possesses German at the academic level. This first translation was back translated into German and has been further revised by an independent translator for both Vietnamese and German. The questionnaire was examined and edited by Vietnamese psychiatrists at the Hospital 103, Hanoi, to ensure that the questionnaire matches the local dialect of Hanoi and to adapt culture-specific help-seeking behaviours within the Vietnamese context.

### Statistical analysis

Descriptive statistics were used to analyze the number of times each treatment option was recommended. Additionally, Chi-squared tests were applied to control for possible demographic differences regarding gender, age and school education between both subsamples. These analyses were separately calculated for each vignette (depression and schizophrenia). Differences in the likelihood of recommendations between both vignettes were analyzed with independent sample t-tests performed on a single item level. A hierarchy of treatment recommendations was determined by estimated means.

## Results

Table 2 displays the frequencies of each category in percentage, mean values and possible mean differences using independent t-tests and 95% confidence intervals for all nine treatment measures. Mean values of all items yielded the same hierarchy of treatment measures recommendations for both conditions. Interestingly, the four most prominent treatment recommendations were similar for both vignettes, namely: psychotherapy, concentration or relaxation exercises, meditation or Yoga and psychotropic medication. The same five treatment measures, natural medicine, ECT, acupuncture, Feng Shui and praying were unfavored for both vignettes. Psychotherapy displayed significantly higher rates of treatment recommendations for patients with schizophrenia in comparison to patients with depression. For the schizophrenia vignette, respondents more strongly recommended and less often discouraged the use of ECT, Feng Shui and praying compared to the depression vignette. For five of the nine items, such as concentration and relaxation exercises, meditation or yoga, psychotropic medication, natural medicine, and acupuncture, there were no significant differences in treatment recommendations between both vignettes.

**Table 2:** Frequencies of treatment recommendation in percentage with means and mean difference with 95% confidence intervals and level of significance for each vignette and treatment measure

Vignette	Response category	Schizophrenia		Depression		Mean differences with CI’s	T-test
Treatment measures		%^a^	Mean^a^	%^b^	Mean^b^		
Psychotherapy	Recommend	92.0		79.4		− 0.308 (− 0.421; − 0.196)	p < .001
	Undecided	7.3	1.60	15.3	1.91		
	Unfavorable	0.8		5.3			
Concentration and relaxation exercises	Recommend	67.7		68.7		− 0.018 (− 0.163; 0.127)	n. s.
	Undecided	21.7	2.24	19.1	2.26		
	Unfavorable	10.6		12.2			
Meditation or Yoga	Recommend	61.0		60.0		0.024 (− 0.126; 0.174)	n. s.
	Undecided	25.9	2.42	27.9	2.40		
	Unfavorable	13.1		12.1			
Psychotropic medication	Recommend	60.3		56.9		− 0.089 (− 0.264; 0.086)	n. s.
	Undecided	20.4	2.46	20.7	2.55		
	Unfavorable	19.3		22.4			
Natural medicine	Recommend	17.6		18.4		− 0.049 (− 0.209; 0.111)	n. s.
	Undecided	44.7	3.36	41.0	3.41		
	Unfavorable	37.7		40.6			
ECT	Recommend	27.7		18.8		− 0.273 (− 0.489; − 0.058)	p < .05
	Undecided	24.5	3.40	23.9	3.67		
	Unfavorable	47.9		57.3			
Acupuncture	Recommend	15.9		16.8		− 0.036 (− 0.199; 0.127)	n. s.
	Undecided	40.3	3.50	35.7	3.53		
	Unfavorable	43.8		47.5			
Feng Shui	Recommend	13.0		11.5		− 0.232 (− 0.406; − 0.057)	p < .01
	Undecided	28.4	3.81	29.4	4.04		
	Unfavorable	58.6		69.1			
Praying	Recommend	13.8		7.1		− 0.333 (− 0.501; − 0.165)	p < .001
	Undecided	26.1	3.85	23.9	4.18		
	Unfavorable	60.1		69.0			

Means defined by a 5-point Likert scale with anchor points from 1 = strongly recommend to 5 = never recommend

^a^Vignette schizophrenia

^b^Vignette depression

Table 3 displays the frequencies of each category in percentage, mean values and possible mean differences using independent t-tests and 95% confidence intervals for all personal and institutional help-seeking options. For the schizophrenia vignette, psychiatrists were the most frequently recommended personal and institutional treatment option, followed by psychotherapists, general practitioner, a person of trust, rehabilitation facility, health authority, and self-help group. For the depression vignette, both a person of trust and a psychotherapist were endorsed most frequently by around 80% of respondents, followed by psychiatrists, general practitioners, rehabilitation facility and strengthening self-reliance. Psychiatrists and psychotherapists were more often endorsed by respondents who had read the schizophrenia vignette compared to the depression vignette. In contrast, a person of trust and doing something by oneself were more often recommended for the depression vignette. For patients with schizophrenia, seeking natural health healer was dissuaded less often compared with the vignette condition depicting depression. In contrast, for patients with depression seeking help via the Internet was less dissuaded compared with patients with schizophrenia.

**Table 3:** Frequencies of recommendations where to seek help with means, mean difference with 95% confidence intervals and level of significance for each vignette and personal or institutional help-seeking option

Vignette	Response category	Schizophrenia		Depression		Mean differences with CI’s	T-test
Personal or institutional treatment option		%^a^	Mean^a^	%^b^	Mean^b^		
Psychiatrist	Recommend	81.9		68.3		− 0.336 (− 0.492; − 0.179)	p < .001
	Undecided	11.6	1.80	18.2	2.13		
	Unfavorable	6.5		13.3			
Psychotherapist	Recommend	81.8		79.4		− 0.149 (− 0.280; − 0.019)	p < .05
	Undecided	14.2	1.82	11.3	1.97		
	Unfavorable	4.0		9.3			
General practitioner	Recommend	74.6		70.5		− 0.069 (− 0.218; 0.081)	n. s.
	Undecided	17.1	2.03	18.6	2.10		
	Unfavorable	8.3		10.9			
Person of trust	Recommend	71.8		81.3		0.158 (0.033; 0.283)	p < .05
	Undecided	22.2	2.14	15.0	1.98		
	Unfavorable	6.0		3.7			
Rehabilitation facility	Recommend	60.2		64.1		0.102 (− 0.040; 0.244)	n. s.
	Undecided	26.8	2.44	26.3	2.33		
	Unfavorable	13.0		9.6			
Health authority/department	Recommend	47.8		47.5		− 0.042 (− 0.214; 0.130)	n. s.
	Undecided	27.5	2.67	28.6	2.71		
	Unfavorable	24.7		23.9			
Self-help group	Recommend	48.7		56.9		0.139 (− 0.035; 0.313)	n. s.
	Undecided	27.3	2.67	22.8	2.53		
	Unfavorable	24.0		20.3			
Self	Recommend	47.3		63.2		0.382 (0.180; 0.583)	p < .001
	Undecided	22.5	2.80	19.2	2.42		
	Unfavorable	30.2		17.6			
Internet	Recommend	26.5		31.4		0.206 (0.027; 0.385)	p < .05
	Undecided	32.4	3.30	35.9	3.10		
	Unfavorable	41.1		32.7			
Natural health healer	Recommend	10.6		6.0		− 0.301 (− 0.460; − 0.142)	p < .001
	Undecided	15.0	4.17	9.4	4.47		
	Unfavorable	74.4		84.6			
Priest	Recommend	7.9		5.6		− 0.145 (− 0.298; 0.008)	n. s.
	Undecided	15.8	4.25	12.9	4.40		
	Unfavourable	76.3		81.5			
Feng Shui master	Recommend	6.3		4.9		− 0.095 (− 0.243; 0.053)	n. s.
	Undecided	13.1	4.38	11.1	4.47		
	Unfavorable	80.6		84.0			
Contact ancestor via medium	Recommend	3.4		3.4		− 0.056 (− 0.184; 0.071)	n. s.
	Undecided	11.3	4.54	9.2	4.60		
	Unfavorable	85.3		87.4			

Means defined by a 5-point Likert scale with anchor points from 1 = strongly recommend to 5 = never recommend

^a^Vignette schizophrenia

^b^Vignette depression

## Discussion

This is the first study to investigate public attitudes and perceptions of treatment recommendations for depression and schizophrenia in Vietnam. Understanding treatment recommendations, as well as response behavior in the context of its sociocultural environment could lead to a deeper understanding of public awareness and causal beliefs of mental health disorders and perceived demands for mental health care provision. Numerous studies highlighted the influence of people’s help-seeking behavior [26, 43] and associated stigma [44] on the course of mental illness.

Remarkably, for both disorders, the same top five recommendations for healthcare providers (psychiatrist, psychotherapist, general practitioner, a person of trust, and rehabilitation facility) received predominately positive recommendations. Moreover, the same five options (internet, natural health healer, priest, Feng Shui master, contact ancestor via a medium) were overall marginally recommended for both conditions. Psychiatrists were among the four most preferred choices for both disorders which are in line with recent research constituting public trust and preferences towards psychiatrists in Vietnam [27]. Interestingly, in Vietnam, the most favored healthcare provider, psychotherapists, received equally high recommendation rates compared to a recent meta-analysis in different Western as well as Eastern societies [37] which reported that 76% recommended psychotherapy for depression and 85% for schizophrenia. This advanced public understanding of adequate psychiatric-psychological treatment, however stands in contrast to the lack of mental health care facilities and integrated psychotherapeutic options in Vietnam. Despite pilot efforts of a study programme of clinical psychology and psychotherapy [45, 46], there is a need for a structured psychotherapy curriculum in Vietnam. This need becomes especially evident when considering that psychotherapy receives the highest recommendation rates of 92% for schizophrenia and 79.4% for depression. However, national guidelines for health insurance have not yet defined psychotherapy, and therefore, health insurances do not cover its cost [47]. Henceforth, the image of a psychotherapist as a person of reference, trust as well as openness and nurturing care and mindful listener seems anchored in society even though its availability and accessibility are lacking [37]. In a next step, an emic public understanding of what psychotherapy defines may be studied with qualitative methods.

Meanwhile, the absence of sensitive sociocultural research on psychotherapeutic interventions in Vietnam remains challenging [14, 15], even though the results indicate that the Vietnamese public primarily endorse professional help. Within the psychiatric outpatient clinic specialized on Vietnamese migrants in Germany at the Charité-Universitätsmedizin further enhanced approaches of informed cultural psychotherapy for Vietnamese patients are in development. Considering the focus on the socio-cultural dimensions of emotion, affect and relationality, such group therapy options show promising results and may be implemented in future studies in Vietnam [31]. Nevertheless, the fact that the four most recommended providers for both vignettes, such as psychiatrist, psychotherapist, a person of trust and a general practitioner, are well-validated interventions in the mental health care system [48–50], suggests a public understanding concerning the complex nature of both mental disorders, schizophrenia and depression. It further indicates an informed mental health literacy of Vietnamese respondents in our sample, manifested in a general trust in a medical approach and psychiatric treatment options, despite its ubiquitous and challenging treatment gap. Additionally, in the context of Vietnam where specialist resources are scare, an approach to tackle the lack of adequate mental health care might be to train general practitioner with basic psychiatric and psychotherapeutic knowledge as a short-time approach while reinforcing the personal resources of the mental health system in the long term.

Even though the public’s recommendation hierarchies are overall similar for both conditions regarding treatment providers, medical professionals, such as psychiatrists, psychotherapists and general practitioners were more strongly endorsed for schizophrenia, while interpersonal-based treatments by a psychotherapist or a person of trust received the highest endorsement for depression. This difference in preferences for treatment options and professional health care providers for both conditions illustrates a public perception that schizophrenia is seen predominantly as a medical and thus biological disorder. In various studies, schizophrenia was perceived as the more severe mental disorder [51–53] followed by alcohol abuse, anxiety disorders and lastly depression, a perceived hierarchy that persists also in the Vietnamese context [22]. Contrasting to schizophrenia, causes of depression, especially in the Asian cultural sphere, were perceived as being rooted in psychosocial or interpersonal related stressors rather than in biochemical dysbalances, [22, 54–56], thus interpersonal treatment providers were more often favoured. These differences between causal beliefs about schizophrenia and depression could also account for the substantial difference in recommendation between schizophrenia and depression for psychiatrists in Vietnam. Moreover, psychiatrists who are licensed to prescribe psychotropic medication might receive attribution of higher competence in dealing with schizophrenia, perceived as a biological illness, rather than a psychological misbalance of mood as in depression [57].

At the same time, self-help strategies and person of trust were recommended more frequently for the depression than for the schizophrenia vignette. Chen and colleagues [54] reported that 75% of Asian American participants with depression endorsed self-management as favorable over pharmacological interventions. The authors also embedded the findings in the understanding of the etiology of depression as being of psychosocial rather than biochemical origin [54]. Furthermore, it was found that relationship-based stressors, such as romantic love or partnership conflicts, were endorsed more frequently as causes of depression in Asian Americans [54, 58, 59], which in the public perception may demand personal advice from a person of trust. A more frequent recommendation of self-help might additionally reflect the perceived value of self-efficacy in the case of depression in contrast to schizophrenia. Endorsing self-efficacy our results showed significantly more respondents, who recommended help-seeking in internet in case of depression in comparison to the schizophrenia vignette condition.

Traditional Vietnamese Medicine (TVM) is deeply rooted in the Vietnamese health system and has nowadays become a part of the formal health care system [60]. In Vietnam, approximately 30% of all patients receive treatment with traditional medicines [60]. Especially in the health prevention and treatment of chronical disease conditions, such as stomach and intestinal disease, Gout or musculoskeletal conditions such as a chronic backache or arthritis TVM plays an important role [61]. However, the same study assessed that only 2.1% of participants used complementary and alternative medicine for the treatment of mental disorders [61]. However, Vietnamese patients with a major depression frequently reported pain or dizziness and patients with “Phong thấp” or “neurasthenia” in the Vietnamese context frequently displayed depressive symptoms [62]. It could be hypothesized, that a part of the patients with chronical illness conditions also has a mental disorder and still used TVM. Interestingly, in our sample, except meditation or Yoga, traditional medicine treatment options such as natural medicine, acupuncture and natural healers were mostly considered unfavourable. A possible explanation is that we used a vignette for depression that did not feature any somatic symptoms. Therefore, the respondents did not associate the case and the use of TVM. The fact that natural healers and treatment measures such as Feng Shui and praying received more endorsement for schizophrenia might be attributed to a state of helplessness in the face of the perceived severity of the disease. Others might turn to supernatural powers or have hope in miracle healings contributing to patient improvement [63] and ultimately inducing a feeling of being in control [64]. Furthermore, schizophrenia symptoms such as thought control, hallucinations, and delusions of reference are more often related to spiritual terms such as possessions and bewitchment [65] rather than interpersonal symptom expressions as in the depression vignette. Thus, in line with symptom perception and causal explanations, spiritual associated treatment facilitators might be more recommended in cases of schizophrenia. In contrast, the effectiveness of traditional therapy options in depression and stress-associated disorders is often higher in comparison to patients affected by psychosis [66]. Moreover, traditional healers who suggest a medication-free approach may influence the course of psychosis by increasing the risk of relapses [67]. However, traditional healthcare providers could also facilitate mental health care utilization in some cultures, if they act as an additional treatment option within the mental health system [68]. Another perspective might be that the utilization of traditional approaches represents a shortage or high barrier of adequate treatment option rather than distrust into medical psychiatry itself [61, 68].

Even though recommendations and dissuasion rates are similarly distributed across both conditions regarding treatment measures, significant differences on individual levels of treatment options are observable. On the one hand, endorsements for ECT display higher levels of dissuasion for both disorders, which may be related to public perceptions of ECT as a drastic measure, due to ECTs historic role in psychiatry and enduring misrepresentation in popular media [69, 70]. On the other hand, even though ECT is one of the five least recommend interventions, there is a significant difference between both vignettes. Over 27% of respondents endorsed ECT for schizophrenia while only around 19% by depression. One reason might be the discussed perception of schizophrenia as a more severe mental illness based on biological defects rather than a psychological imbalance of mood [22, 36, 71, 72].

Vietnamese respondents for both vignettes endorsed overall high levels of recommendations for low-threshold interventions, such as relaxation or concentration training, as well as meditation or yoga, which may be perceived as less harmful and non-intrusive. Yoga and meditation are the two measures which are nearly equally frequently recommended for schizophrenia and depression. These results for both conditions stand partially in contrast to findings from Western countries were low-threshold interventions such as meditation, yoga, and acupuncture were declined while relaxation was recommended [73]. This discussion is weighed in the light of Vietnam’s cultural context, which is influenced by Buddhist traditions and folk religions [74]. Concentration and relaxation exercises such as mindfulness, meditation, and yoga are deeply rooted in Buddhist shaped societies and have a long enduring tradition in facilitating mental well-being [75, 76].

The current study must be interpreted in the light of several limitations which should be addressed in future research. First, the study was conducted in the rural and urban areas of Hanoi, the capital city of Vietnam leading to results, which cannot be generalized for the whole country, acknowledging its social and cultural diversity. Second, overall higher educational levels of participants from Hanoi compared to of the population in Vietnam (77.8% with upper secondary school vs. 66.7%) could explain a potentially higher mental health literacy. Third, the collected data was assessed cross-sectionally, limiting any causal explanatory power of the study. Finally, a list of a priori determined answer possibilities might account for bias, as this approach may render further answer options unfeasible. However, sociocultural sensitive treatment facilitators such as acupuncture, seeking Feng Shui master or contact ancestor via medium were considered. Nevertheless, qualitative approaches might give insight into new explanatory patterns, respondents understanding of terms and answer possibilities.

## Conclusion

When summarizing the findings on the publics’ perception of interventions in the treatment of two major mental illnesses, it appears that the Vietnamese public recognizes mostly evidence-based treatment recommendations from a variety of given options and can ascribe them according to a perceived illness severity and treatment need. However, the current inadequate mental health care infrastructure and the lack of psychiatric and psychotherapeutic personnel constitutes a barrier to care provision, even though the public recognizes its importance and feasibility. Thus, any further development of the Vietnamese mental health care system, especially with a focus on building human resources is essential. Moreover, the high recommendation of psychotherapy and consulting psychotherapists indicates the high need for legal regulation of psychotherapy, as well as professional training for therapists and medical doctors and further in-depth research concerning these issues in the sociocultural context of Vietnam.
